# Tissue-specific transcriptome and metabolome analyses reveal candidate genes for lignan biosynthesis in the medicinal plant *Schisandra sphenanthera*

**DOI:** 10.1186/s12864-023-09628-3

**Published:** 2023-10-11

**Authors:** Boshi Sun, Peng Wang, Meng Guan, Entong Jia, Qian Li, Jun Li, Ziyun Zhou, Pengda Ma

**Affiliations:** 1 College of Life Science, Northwest A & F University, No. 22 Xinong Road, Yangling, 712100 Shaanxi China; 2 College of Life Science, Northeast Forestry University, Harbin, 150040 China; 3 Institute of Chinese Materia Medica, China Academy of Chinese Medical Sciences, Beijing, 100700 China

**Keywords:** *Schisandra sphenanthera*, Targeted metabolome, Transcriptome, Lignan biosynthesis, Weighted gene co-expression network analysis

## Abstract

*Schisandra sphenanthera* is an extremely important medicinal plant, and its main medicinal component is bioactive lignans. The *S. sphenanthera* fruit is preferred by the majority of consumers, and the root, stem, and leaf are not fully used. To better understand the lignan metabolic pathway, transcriptome and metabolome analyses were performed on the four major tissues of *S. sphenanthera*. A total of 167,972,229 transcripts and 91,215,760 unigenes with an average length of 752 bp were identified. Tissue-specific gene analysis revealed that the root had the highest abundance of unique unigenes (9703), and the leaves had the lowest (189). Transcription factor analysis showed that MYB-, bHLH- and ERF-transcription factors, which played important roles in the regulation of secondary metabolism, showed rich expression patterns and may be involved in the regulation of processes involved in lignan metabolism. In different tissues, lignans were preferentially enriched in fruit and roots by gene expression profiles related to lignan metabolism and relative lignan compound content. Furthermore, schisandrin B is an important compound in *S. sphenanthera*. According to weighted gene co-expression network analysis, PAL1, C4H-2, CAD1, CYB8, OMT27, OMT57, MYB18, bHLH3, and bHLH5 can be related to the accumulation of lignans in *S. sphenanthera* fruit, CCR5, SDH4, CYP8, CYP20, and ERF7 can be related to the accumulation of lignans in *S. sphenanthera* roots. In this study, transcriptome sequencing and targeted metabolic analysis of lignans will lay a foundation for the further study of their biosynthetic genes.

**Supplementary Information:**

The online version contains supplementary material available at 10.1186/s12864-023-09628-3.

## Introduction

*Schisandra sphenanthera* Rehd. et Wils (*S. sphenanthera*) is a single species of perennial deciduous scandent woody vine plant of *Schisandra* genus (Magnoliaceae) and is a horticultural plant with edible fruit [1, 2]. The fruit of *S. sphenanthera* is also known as “Huazhongwuweizi” or “Nanwuweizi” in Chinese according to the 2020 Edition of Chinese Pharmacopoeia [3] because it is mainly grown in the middle and south areas of China, such as Gansu, Shaanxi, Shanxi, Henan, Shandong, Yunnan, Sichuan, Guizhou, Hunan, Hubei, Anhui, Jiangsu, Zhejiang, Jiangxi, and Fujian [4–7]. It is a famous Traditional Chinese Medicine and was first recorded in the Chinese “Shen Nong Ben Cao Jing,” with a history of more than 2000 years. Since 2000, it has been officially included in the Chinese Pharmacopoeia as a tonic, antitussive, and sedative agent [8]. The World Health Organization has also listed it in the International Pharmacopoeia [9]. In addition, *S. sphenanthera* fruit has been recognized by the Ministry of Health of the People’s Republic of China as one of the materials than can be used in health products, cosmetics, and functional food [10]. For example, the fruit is consumed in a variety of ways as tea, fruit wine, yogurt, and food additives [11]. Given that the nutritious and delicious *S. sphenanthera* fruit has the characteristics of medicine food homology, it is widely popular to the majority of consumers. However, owing to the increase in the world’s population and despite improvements in human living and medicine, relying only on the fruit as a pharmaceutical raw material is insufficient to meet people’s needs. Therefore, fully exploiting the other tissues of *S. sphenanthera*, such as roots, stems, and leaves, is of great significance and has huge market potential.

*S. sphenanthera* is generally used to treat diabetes, hepatitis, hepatosis, chronic cough, insomnia, and menstrual disorders [12–14] owing to its hepatoprotective [15], renoprotection [16], cardioprotection [17], neuroprotection [18], antioxidant [19], anticancer [11], anti-inflammatory [20], antiviral [21], anti-osteoporosis [22], anti-HIV [11], anti-COVID-19 [23], and immunomodulatory [24] effects and other activities [7]. These features are due to the numerous active substances of *S. sphenanthera* extracts. To date, 310 active substances have been isolated and identified from this plant, mainly lignans with the dibenzocyclooctadiene skeleton or coniferol dimer basic structure, which are usually referred to as C6-C3 unit compounds [10, 25]. Given that lignans are only present in this particular species, these biologically active substances are commonly referred to as wuweizi lignans or schisandra lignans, such as schisandrol A, schizandrol B, schisantherin A, schisantherin B, schisandrin A, schizandrin B, schisandrin C, and anwulignan [26, 27]. Furthermore, some other substances, such as triterpenes, sesquiterpenes, flavonoids, phenolic acids, essential oils, organic acids, vitamins, amino acids, and polysaccharides [3, 13, 28, 29] are also found in these plants and contribute to its biological activities. Although lignans play an essential role in *S. sphenanthera*, its distribution and regulation in the root, stem, leaf, and fruit is still unclear.

Schisandra lignans have broadly pharmacological activities, such as anti-hepatitis, anti-inflammatory, antioxidant, and anti-HIV, improving insulin sensitivity [3, 10, 30], so exploring its biosynthesis is of great significance. The biosynthesis of lignans is roughly divided into three stages. The first stage is the phenylpropanoid metabolic pathway. First, phenylalanine ammonialyase (PAL) catalyzes the deamination of phenylalanine to form cinnamic acid. Then, *ρ*-coumaric acid is generated from cinnamic acid by cinnamate 4-hydroxylase (C4H). PAL and C4H are the key enzymes in the phenylpropanoid metabolic pathway [31]. The second stage is the specific synthesis of lignan monomer. *ρ*-coumaric acid is finally converted into lignan monomer (coniferyl alcohol) by *ρ*-coumarate 3-hydroxylase (C3H), 4-coumarate CoA ligase (4CL), caffeic acid 3-*O*-methyltransferase, caffeoyl CoA-*O*-methyltransferase (CCoAOMT), cinnamoyl CoA-reductase (CCR), and cinnamyl alcohol dehydrogenase (CAD) [25]. The third stage is the polymerization of lignan monomers. Coniferyl alcohol is oxidized into lignan polypolymer under the action of dirigent (DIR). Finally, the lignan polymer is further transformed into a variety of lignan by pinoresinol lariciresinol reductase (PLR), pluviatolide synthase (PLS), and secoisolariciresinol dehydrogenase (SDH) [25, 32]. Although the biosynthesis of lignans has been widely studied, the molecular mechanisms responsible for the biosynthesis and regulation of lignans in *S. sphenanthera* remain unclear.

In this research, transcriptomes in the four tissues of *S. sphenanthera* were de novo sequenced and assembled using the PE150 sequencing technology of the Illumina platform. The expression profiles of genes related to the lignan biosynthesis pathway in *S. sphenanthera* were obtained. By comparing the transcriptome and tissue-specific metabolite accumulation, the molecular mechanism insights into the biosynthesis and regulation in *S. sphenanthera* was revealed. We identified genes for enzymes involved in lignan synthesis and determined the relative expression of these genes in different tissues. In conclusion, this study not only provides a theoretical basis for molecular marker-assisted breeding of *S. sphenanthera*, but also lays a foundation for the biosynthesis and regulation of active substances in different tissues. Our work provides a valuable resource for research into metabolic engineering in *S. sphenanthera*, an important medicinal plant.

## Materials and methods

### Plant materials

*S. sphenanthera* was identified by Professor Liang Zhao of the College of Life Science, Northwest A & F University, and stored in the specimen library of Northwest A&F University (ID: ZS202204001). *S. sphenanthera* samples were cultured in the Zuoshui Century Ecological Agriculture Co., Ltd. in Shangluo, Shaanxi, China (33° 41′ 8′′ north latitude, 109° 17′ 32′′ east longitude). Fresh tissue samples (roots, stems, leaves, and fruit) were collected from three-year-old plants in 2022. Healthy tissue samples were cleaned three times with distilled water, immediately frozen in liquid nitrogen, and stored at − 80 °C. Samples of the same tissue from three independent plants of *S. sphenanthera* were mixed.

### Total RNA extraction and transcription sequencing

Total RNA was extracted from the root, stem, leaf, and fruit tissues from three individual plants with RNA prep pure polysaccharide polyphenol plant total RNA extraction kit (DP441, Tian gen Biotechnology Co., Ltd, Beijing, China) and repeated three times. The concentrations, integrity, and purity of the RNA samples were examined using a NanoPhotometer spectrophotometer (IMPLEN, Munich, Germany), Agilent 2100 system (Agilent Technologies Inc, CA, USA), and 1% agarose gel electrophoresis system, respectively. RNA sequencing libraries with insert sizes ranging from 250 to 300 bp were constructed. RNA sequencing was performed on an Illumina Novaseq 6000 (Illumina, CA, USA) with paired-end reads of 150 bp. After high-quality sequencing data were obtained, TRINITY (version 2.11.1) was used to assemble the sequences and obtain the transcript sequences [33]. Finally, redundant transcripts were removed to collect the unigenes.

### Gene function annotation and classification

To predict gene function, unigenes were annotated with NR (NCBI non-redundant protein sequences), SwissProt (a manually annotated and reviewed protein sequence database), Gene Ontology database (GO), Clusters of Orthologous Groups of protein database (COG), and Kyoto Encyclopedia of Genes and Genomes pathways database (KEGG). the BLAST program was used with an *E*-value of 1e-5. When the results of the different databases conflict, the SwissProt database was selected first, followed by the NR, KEGG, and COG databases.

### Differential gene expression analysis

Salmon v1.2.29 with default parameters estimated gene expression levels in each sample according to fragments Per kilobase of transcript per million mapped reads (FPKM). Differentially expressed genes (DEGs) between each pair of samples were performed using DESeq R package. Fold-change values between samples were estimated on the basis of the FPKM values. The |log2(fold change)| value > 1, and p value < 0.05 was used as the threshold to evaluate the significance level of differential gene expression.

### Transcription factor analysis

To research the transcription factor families in *S. sphenanthera*, the transcripts were mapped for all transcription factor protein sequences available in the Plant Transcription Factor Database (PlantTFDB v. 4.02) using BLAST, with an *E*-value threshold of 1e-5.

### Targeted metabolome analysis

Standards and solvents: Anwulignan (CAS: 107534-93-0), schisandrin A (CAS: 61281-38-7), schisandrin B (CAS: 61281-37-6), schisandrin C (CAS: 61301-33-5), schisandrol A (CAS: 7432-28-2), schisandrol B (CAS: 58546-54-6), schisantherin A (CAS: 58546-56-8), and schisantherin B (CAS: 58546-55-7) were used as external standards and purchased from BioBioPha Co., Ltd. Penicillin G (CAS: 69-57-8) was used as an internal standard and purchased from Shanghai Acmec Biochemical Co., Ltd. All the solvents, such as methanol (CAS: 67-56-1, Thermo Fisher Scientific), were of chromatography grade. Watsons water was used. Formic acid (CAS: 64-18-6) was purchased from Thermo Fisher Scientific.

Sample preparation: Metabolites were extracted from the same tissues as those used in RNA sequencing analysis for the relative quantitative analysis of lignan, and three biological replications were established. All the samples were freeze dried, crushed, and sieved through a No. 20 mesh sieve. Powder (100 mg) was extracted with 1 mL of 70% methanol water (containing 5 µg/mL Penicillin G) at 4 °C overnight and subjected to ultrasonication for 30 min. After centrifugation at 12 000 ×g for 10 min, the extracts were collected and filtrated using a 0.20 μm microporous membrane (Agilent Technologies, Santa Clara, CA, USA).

Appropriate amounts of eight standards were weighed and dissolved in methanol for the preparation of 1 mg/mL stock solutions. Each stock solution was mixed in methanol for the preparation of the quality control (QC) sample comprising 10 µg/mL anwulignan, schisandrin A, schisandrin B, schisandrin C, schisandrol A, schisandrol B, schisantherin A, and schisantherin B. QC samples were placed in the queue for every five experimental samples and used in determining system suitability and providing a means of monitoring the reproducibility and stability of the LC/MS system.

LC/MS analysis: Metabolites were identified and quantified using the Agilent UPLC 1290 system combined with a G6500 quadrupole time-of-flight mass spectrometer (QTOF, Agilent Technologies, Santa Clara, CA, USA) and G6400 triple quadrupole mass spectrometer (QQQ, Agilent Technologies, Santa Clara, CA, USA). QTOF and QQQ use an electrospray ionization source. Metabolite identification was determined according to retention time and the mass spectra of the positive ion modes of QTOF by standard substance and public databases. Public databases included MassBank, ReSpect, mzCloud, KNAPSAcK, and HMDB. Metabolite quantification was carried out using multiple-reaction monitoring (MRM) of QQQ in positive ion ([M + H]^+^) modes. The MS scan functions and UPLC solvent gradients were controlled by Agilent MassHunter Workstation software. The analytical conditions were as follows: Agilent Eclipse Plus C_18_ column, 2.1 mm × 150 mm, 1.8 μm; injection volume, 3 µL; temperature, 35 °C; flow rate, 0.3 mL/min; detection wavelength, 245 and 254 nm; and solvent system, water containing 0.1% formic acid (A) and methanol (B). The separation gradient was as follows: 5% phase B (0–11.0 min), 70% phase B (11.0–17.0 min), 75% phase B (17–23.0 min), 95% phase B (23.0–23.1 min), 95% phase B (23.1–28.0 min), and phase B (28.0–30.0 min).

### Weighted gene co-expression network analysis

Weighted gene co-expression network analysis (WGCNA) was performed to assess the gene co-expression networks associated with lignan content in *S. sphenanthera* by using the R (version 4.2.1). The genes with FPKM > 10 were selected for WGCNA with the following settings: CV values, < 0.5; minimum module size, 30; and minimum height for merging modules, 0.25.

### qRT-PCR analysis

Total RNA was extracted using the Steady Pure Universal RNA Extraction Kit II (AG21022, Accurate Biotechnology, China), and cDNA was synthesized using the Evo M-MLV RT mix kit with gDNA clean for qPCR (Ver. 2; AG11728; Accurate Biotechnology, China). Gene primers were designed using Primer3web (https://primer3.ut.ee/; Table S15). The glyceraldehyde-3-phosphate dehydrogenase gene was used as the internal reference gene [34]. Cham Q SYBR qPCR Master Mix (Vazyme Biotech Co., Ltd, China) was used for the RT–PCR reactions. The reaction mixture contained 7.5 µL of 2× Cham Q SYBR qPCR Master Mix, 1.0 µL of cDNA, 1.0 µL of primers (10 µM), and 4.5 µL of ddH_2_O. The qRT-PCR reaction parameters were 95 °C for 30 s, 40 cycles of 95 °C for 10 s, and 60 °C for 20 s. Relative gene expression was calculated using the 2^−ΔΔCT^ method [35]. Three replicate measurements were performed on each sample.

### Statistical analysis

All the experiments were conducted in duplicate. The results were analyzed using GraphPad Prism 5.0 (GraphPad Inc., La Jolla, CA, USA) and one-way analysis of variance (SPSS 21.0; IBM Corp., Armonk, NY, USA). Mean differences were compared using student t-tests, with a significant level of < 0.05.

## Results

### RNA sequencing and de novo transcriptome assembly

**Fig. 1:**
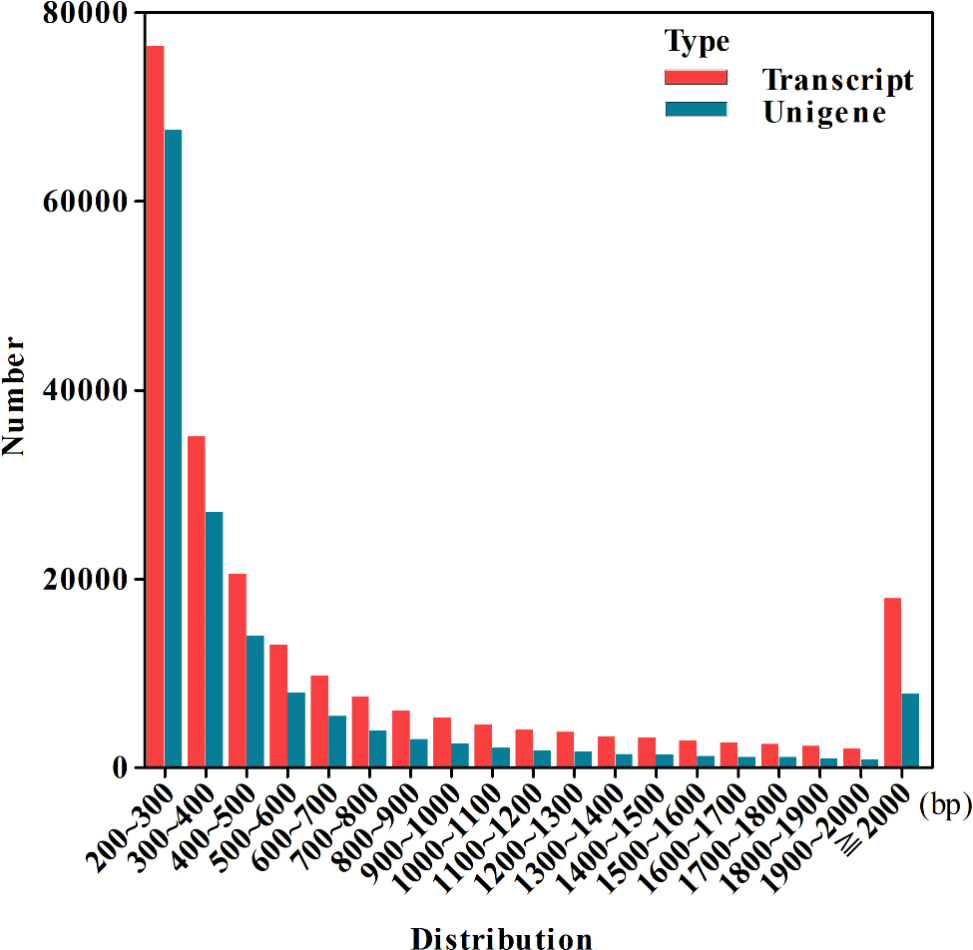
Length distribution of the assembled transcripts and unigenes

The transcriptomes of each of the four tissues were produced by Illumina Sequencing Technology. The raw reads obtained by the RNA sequencing were processed. A total of 318,938,560 (92.68 G bases) high-quality sequencing reads were obtained after removing low-quality and incorrect reads from 333,733,702 (100.12 G bases) raw reads. As shown in Table S1, the quality of the reads of different tissues was provided. The total number, minimum length, maximum length, mean length, N50, and N90 of the transcripts and unigenes are summarized in Table S2. The N50 of the transcript was 1304 bp, the N90 of the transcript was 284 bp, its minimum length was 186 bp, its maximum length was 14,668 bp, and its mean length was 752 bp. These results indicated the assembly of high-quality transcriptomes in this study. To obtain a unique representative transcript for a single gene, the longest transcript was considered a single unigene for each gene regardless of splice variants. A total of 91,215,760 unigenes were assigned from a total of 167,972,229 assembled transcripts (Table S2). The length distributions of the transcripts and unigenes are shown in Fig. 1 and Table S3.

### Functional annotation and classification

**Fig. 2:**
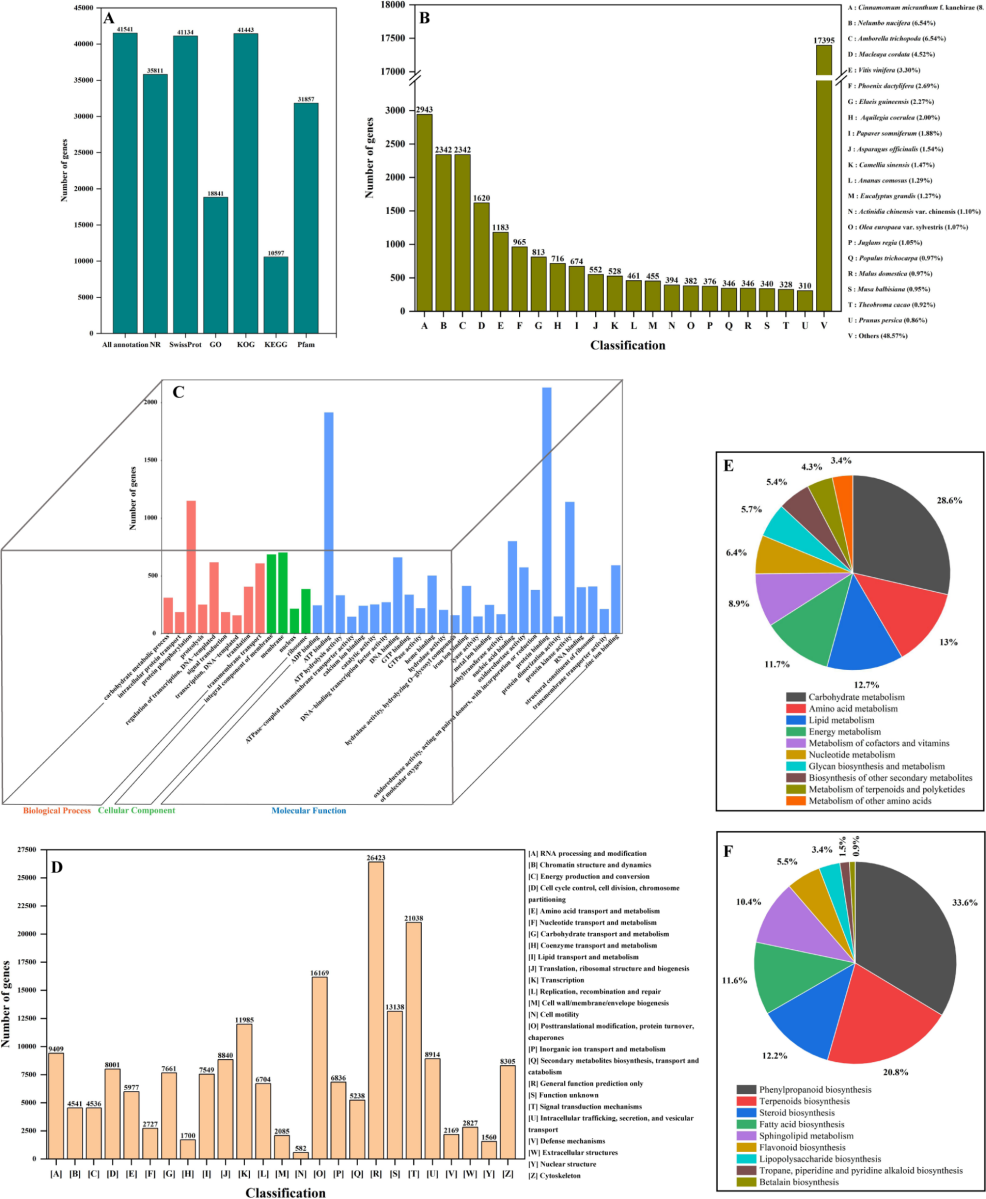
Functional annotation and classification of the predicted unigenes. (A) The number of unigenes annotated according to different resources and databases. Annotation was carried out on the basis of sequence similarity as determined by NCBI BLAST analysis. (B) The pie chart represents the distribution of significant blast hit species with respect to identified unigenes. (C) Gene Ontology classification of the assembled unigenes. (D) KOG classification. (E) KEGG classification. (F) KEGG classification of secondary metabolites

The functional annotation of assembled transcripts provides information about molecular functions and biological processes. Six public databases, namely, NR, Swiss-Prot, GO, KOG, KEGG, and Pfam, were used for the functional annotation of the predicted proteins. A total of 41,541 unigenes (27.09%) were annotated in the public databases (Fig. 2A and Table S4). In addition, 35,811 (23.35%), 41,134 (27.00%), 18,841 (12.29%), 41,443 (27.02%), 10,597 (6.91%), and 31,857 (20.77%) unigenes of *S. sphenanthera* were annotated in the NR, Swiss-Prot, GO, KOG, KEGG, and Pfam databases, respectively (Fig. 2A and Table S4). To obtain isogenous genes among plant species, the annotated unigenes of *S. sphenanthera* were inquired using the NR database. As Fig. 2B shows, the highest proportion of homologous unigenes was found in *Cinnamomum micranthum* f. kanehir (8.22%), followed by the proportions in *Nelumbo nucifera* (6.54%), *Amborella trichopoda* (6.54%), *Macleaya cordata* (4.52%), and *Vitis vinifera* (3.30%; Table S5). The unigenes (48.57%) without a match with those of other species were considered species specific in *S. sphenanthera*.

A total of 21,591 unigenes were identified using GO analysis based on NR annotation, including molecular function (13,687 unigenes), biological process (5289 unigenes), and cellular component (2615 unigenes). In molecular function, the most predominant enrichment was related to protein binding, ATP binding, and protein kinase activity. In biological processes, unigenes were mostly enriched in protein phosphorylation, transcription regulation, and transmembrane transport. In cellular components, unigenes were mostly enriched in the integral components of membranes and ribosomes (Fig. 2C and Table S6). Furthermore, 194,914 unigenes were classified into 25 categories in the KOG database (Fig. 2D and Table S7). Most unigenes were enriched in “general function prediction only” (26,423), followed by “signal transduction mechanisms” (21,038), “post-translational modification, protein turnover, chaperones” (16,169), and “transcription” (11,985).

Most unigenes were involved in metabolism, including carbohydrate metabolism (28.60%), amino acid metabolism (13.00%), lipid metabolism (12.70%), energy metabolism (11.7%), metabolism of cofactors and vitamins (8.90%), nucleotide metabolism (6.40%), glycan biosynthesis and metabolism (5.70%), biosynthesis of other secondary metabolites (5.40%), metabolism of terpenoids and polyketides (4.30%), and biosynthesis of other secondary metabolites (3.40%; Fig. 2E and Table S8). Among other secondary metabolites, the most dominant category was phenylpropanoid biosynthesis (33.60%), followed by terpenoid biosynthesis (20.80%), steroid biosynthesis (12.20%), fatty acid biosynthesis (11.60%), sphingolipid metabolism (10.40%), flavonoid biosynthesis (5.50%), lipopolysaccharide biosynthesis (3.40%), tropane, piperidine and pyridine alkaloid biosynthesis (1.50%), and betalain biosynthesis (0.90%; Fig. 2F and Table S9).

### Analysis of differentially expressed genes

**Fig. 3:**
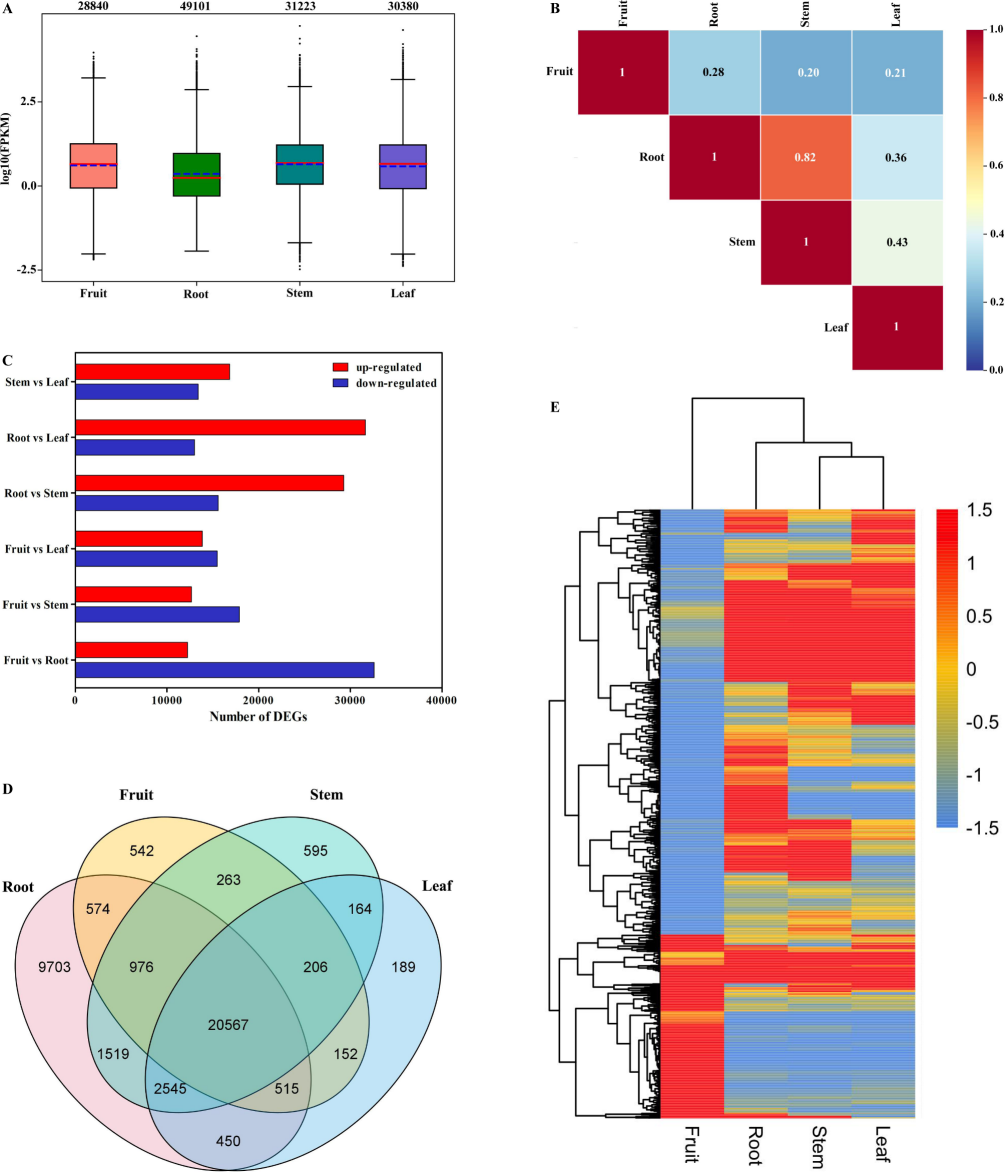
Overview of expressed unigenes, TSGs, and DEGs. (A) Boxplot of unigenes expressed in the four tissues, presenting the distributions of expression levels. (B) The square values of Pearson’s correlation coefficients (R^2^). (C) Number of the DEGs in different comparisons. Red represents up-regulated genes, and blue represents down-regulated genes. (D) Venn diagram showing the overlapping uigenes among tissues. (E) Overall clustering analysis and heat map of the four groups of samples (fruit, root, stem, and leaf,)

In this study, the expression abundance of unigenes were evaluated using FPKM values (Table S10). As shown in Fig. 3, the expressed unigenes, tissue-specific genes (TSGs), and DEGs were presented. The expression levels of unigenes are shown in Fig. 3A, indicating that each cluster had a pronounced stable distribution pattern in each tissue. The expression of 49,299 unigenes were detected in the four tissues (Fig. 3E and Table S10). The square value of Pearson’s correlation coefficient (R^2^) was above 0.8 in three replicates for each tissue, indicating the high reproducibility of the unigene expression. This showed that the correlation between the roots and stems was the highest (0.82), followed by that between the stems and leaves (0.43; Fig. 3B). The number of DEGs between the three different tissues were as follows: 44,838 between fruit and root (27.33% up-regulated and 73.67% down-regulated), 30,566 between fruit and stem (41.44% up-regulated and 58.56% down-regulated), 29,330 between fruit and leaf (47.21% up-regulated and 52.79% down-regulated), 44,886 between root and stem (65.26% up-regulated and 34.74% down-regulated), 44,650 between root and leaf (70.89% up-regulated and 29.11% down-regulated), 30,241 between stem and leaf (55.68% up-regulated and 44.32% down-regulated; Fig. 3C). In all the DEGs, 11,029 unigenes expressed in only one tissue were identified as TSGs, and 20,567 common unigenes were ubiquitously expressed in all four tissues (Fig. 3D and Table S10). The root provided the most number of TSGs (9,703), followed by the stem (595), fruit (542), and leaf (189). Heatmap hierarchical clustering analysis was performed on FPKM value to resolve the global expression patterns of the four tissues (Fig. 3E). The results showed that the unigenes greatly varied among the tissues, and the expression of unigenes in the fruit showed the most pronounced expression pattern.

### Transcription factors

**Fig. 4:**
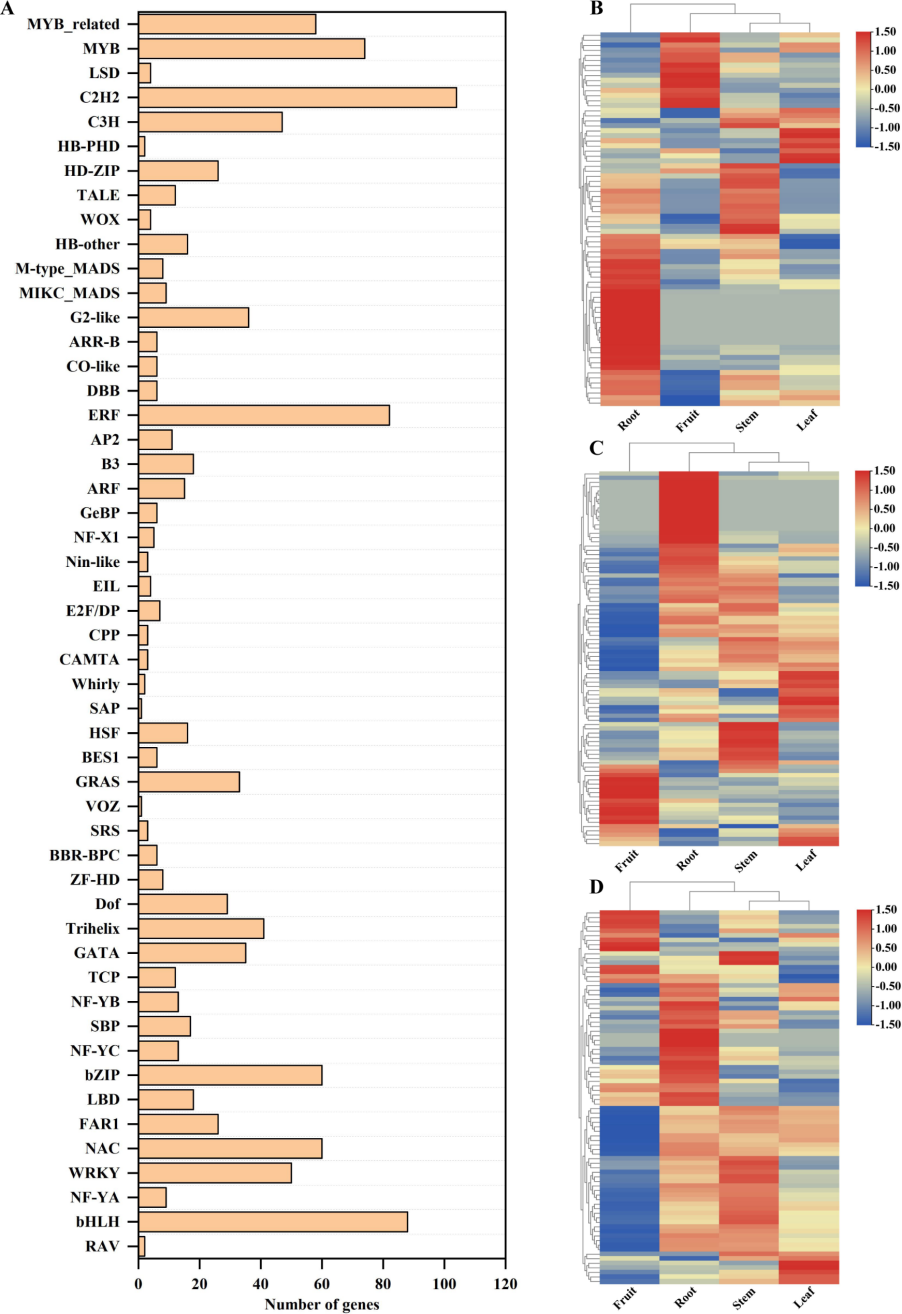
Classification and clustering analysis of putative transcription factor families. (A) Classification of the transcription factors. (B, C, and D) Overall clustering analysis and heatmap of the expression of MYB- (B), bHLH- (C), and ERF- (D) family transcription factors derived in the four samples

Transcription factors are the main regulatory factors for the expression of networks of target genes, developmental processes, and secondary metabolism in plants. In this study, 1124 unigenes were identified as putative transcription factors belonging to 51 transcription factor families. C2H2 (104), the basic helix-loop-helix (bHLH) (88), ethylene responsive factors (ERFs) (82), MYB (74), and WRKY (50) families had a higher number of members (Fig. 4A and Table S11). Many family members of MYB, bHLH, and ERF play important roles in secondary metabolite regulation in plants [36–38]. In *S. sphenanthera*, most MYB, bHLH and ERF unigenes had significantly high expression levels in the roots and showed a unique expression pattern in the fruit (Fig. 4B, C and D, and Table S11). This result suggested that they play an important role in regulating the production of some metabolites.

### Targeted metabolomic profiling of lignans

**Fig. 5:**
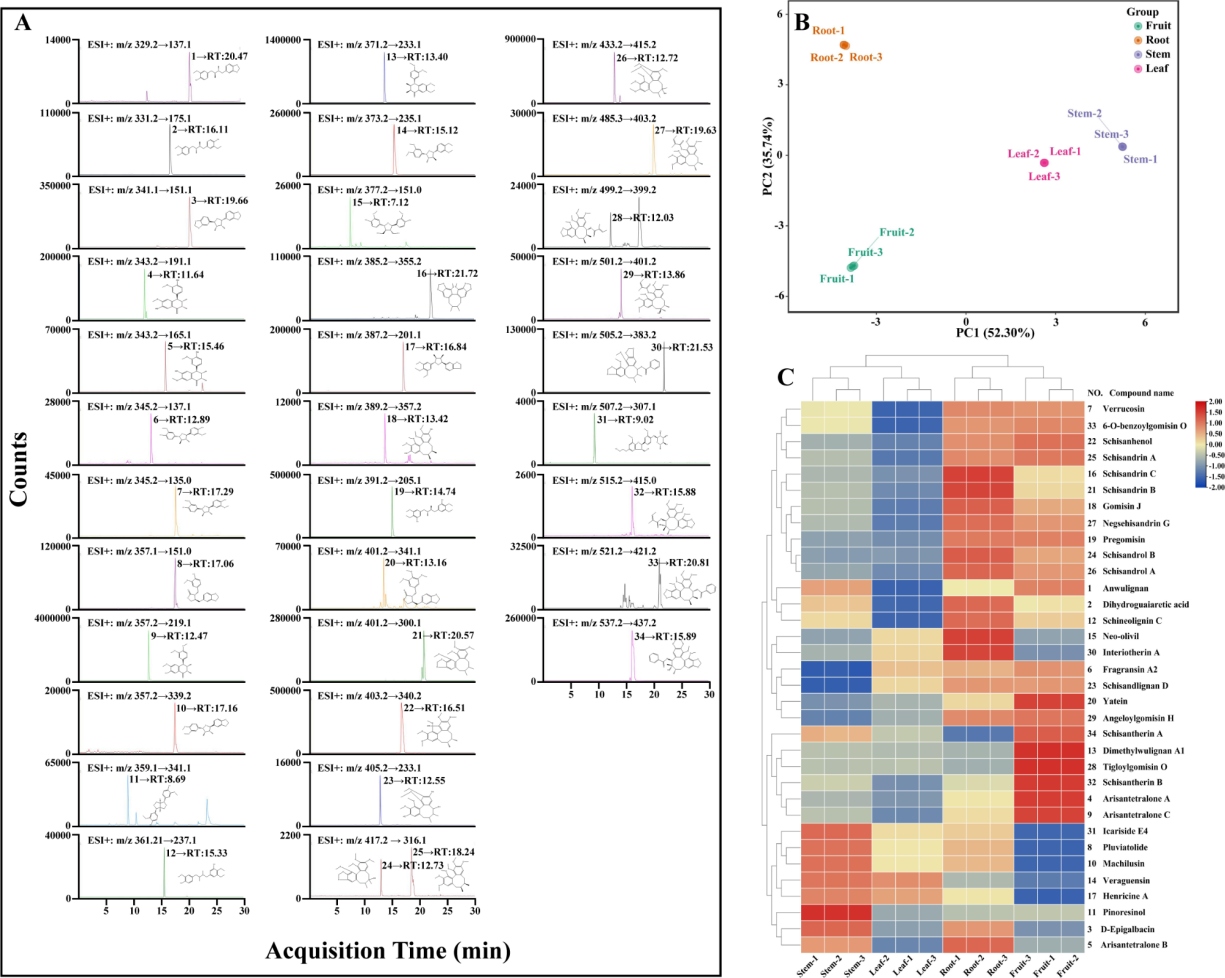
Targeted metabolomics analysis of *S. sphenanthera*. (A) The optimized extracted ion chromatograms of the 34 compounds. RT indicates the retention time, expressed in min. Compound number is consistent with the compound number in Table S12. (B) PCA score plots of lignans in four tissues of *S. sphenanthera*. (C) Heatmap based on hierarchical clustering analysis

A total of 34 lignans were successfully characterized using UPLC–QTOF–MS in *S. sphenanthera* (Table S12). UPLC-QQQ-MS was used to further optimize the collision energy of the lignans (Fig. 5A). Afterward, the levels of the 34 lignan compounds in the four tissues of *S. sphenanthera* were quantified according to the peak areas with the MRM mode. Principal component analysis (PCA) showed that the repeatability within the four tissues was well depicted (Fig. 5B), indicating the steady and reliability of the metabonomic test. The heatmap of hierarchical clustering showed that the accumulation of different lignans in different tissues of *S. sphenanthera* varied considerably (Fig. 5C). In *S. sphenanthera*, the fruit had the highest level of lignan accumulation, followed by roots, stems and leaves. Anwulignan, schisandrin A, schisandrin B, schisandrin C, schisandrol A, schisandrol B, schisantherin A, and schisantherin B are lignan compounds commonly found in *S. sphenanthera*. The findings indicated that the high levels of schisandrin B in the fruit, roots, stems, and leaves can serve as characteristic markers for the quality assessment of *S. sphenanthera*.

### Integration analysis of structural genes and metabolites

**Fig. 6:**
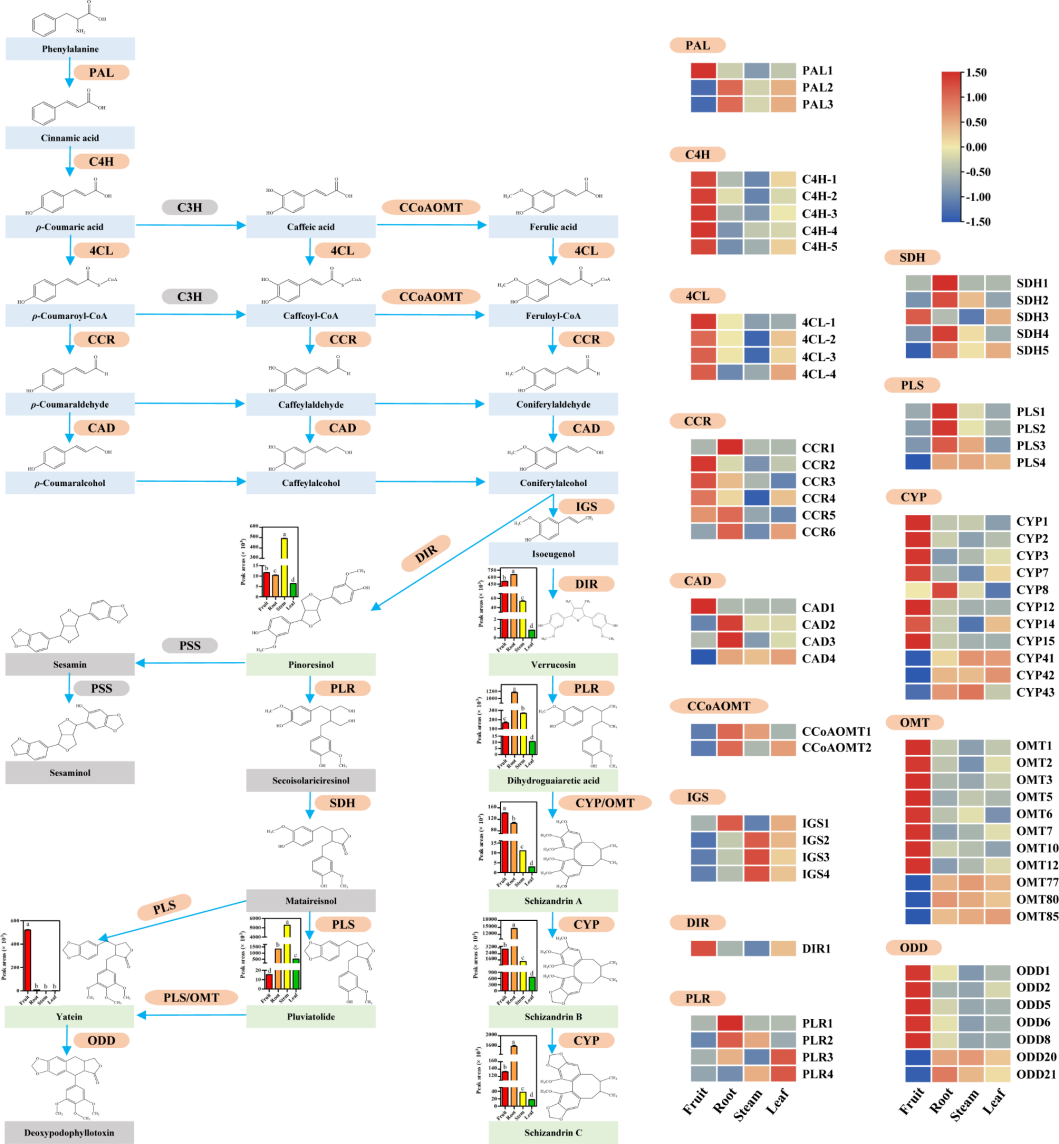
Lignan biosynthetic pathway in the four tissues of *S. sphenanthera*. The heat map shows the fruit, roots, stems, and leaves from left to right. The color scale indicates log2 (reads per kilobase per million mapped read values) for the four tissues. Each color circle represents the level of gene expression according to the color scale. The bar chart illustrates the relative content of lignans in the biosynthetic pathway and shows the fruit, roots, stems, and leaves from left to right. Light blue represents the upstream material. Light green represents lignans detected. Orange represents the detected structural genes. Gray represents undetected substances or genes. The detailed information of 34 differentially accumulated lignans is shown in Table S12. PAL, phenylalanine-ammonia-lyase; C4H, cinnamate-4-hydroxylase; C3H, cinnamate-3-hydroxylase; CCoAOMT, caffeoyl-CoA-O-methyltransferase; 4CL, 4-coumarate-CoA-ligase; CCR, cinnamoyl-CoA-reductase; CAD, cinnamyl-alcohol-dehydrogenase; IGS, isoeugenol-synthase; DIR, dirigent protein; PLR, Pinoresinol/lariciresinol-reductase; CYP, Cytochrome P450; OMT, O-methyltransferase; PSS, Piperitol/sesamin-synthase; SDH, Secoisolariciresinol-dehydrogenase, PLS, Pluviatolide-synthase; ODD, 2-oxoglutarate/Fe(II)-dependent-dioxygenase.

All the structural genes known to be involved in lignan biosynthesis were found in the unigene dataset, including PAL, C4H, 4CL, CCR, CAD, CCoAMOT, IGS, PLR, SDH, PLS, CYP, OMT, and ODD (Table S13). To obtain difference in lignan biosynthesis pathway among the four tissues, we observed the abundances of the 673 unigenes encoding 17 structural genes and identified 34 differentially accumulated lignans (Table S12 and Table S13). The whole lignan biosynthetic pathway can be divided into phenylpropanoid metabolic pathway, lignan monomer synthesis, and lignan monomer polymerization (Fig. 6). The relatively high content of pioresinol and pluviatolide in the stems can be related to C4H-8, C4H-26, C4H-53, 4CL-27, CCR8, CAD5, and CAD20 in the phenylpropanoid metabolic and lignan monomer synthesis pathways. In addition, pluviatolide may be associated with SDH4, SDH5, and PLS4 in lignan monomer polymerization. The relative content of yatein and schizandrin A was high in the fruit, consistent with PAL1, C4H-1, C4H-2, 4CL-4, CCR2, CCR3, and CAD1 in the phenylpropanoid metabolic and lignan monomer synthesis pathways, and OMT1-12 in lignan monomer polymerization. Furthermore, schizandrin A may be related to CYP1-3, CYP7, CYP12, and CYP14-15 in lignan monomer polymerization. The relative content of verrucosin, dihydroguaiaretic acid, schizandrin B, and schizandrin C in the roots were high, possibly related to PAL2-3, CCR1, CCR5-6, and CAD2-3 in phenylpropanoid metabolic and lignan monomer synthesis pathways. IGS1 was related to a specific synthetic pathway involving lignans. Dihydroguaiaretic acid, schizandrin B, and schizandrin C may be related to PLR1-2 in lignan monomer polymerization. Furthermore, schizandrin B and schizandrin C may be related to CYP8, OMT77, OMT80, and OMT85 in lignan monomer polymerization (Fig. 6 and Table S13). If the transcriptional profiles of structural genes encoding the same or different families show different expression patterns, the genes may indicate the other functions of the pathway.

### Putative interaction networks involved in lignan biosynthesis

**Fig. 7:**
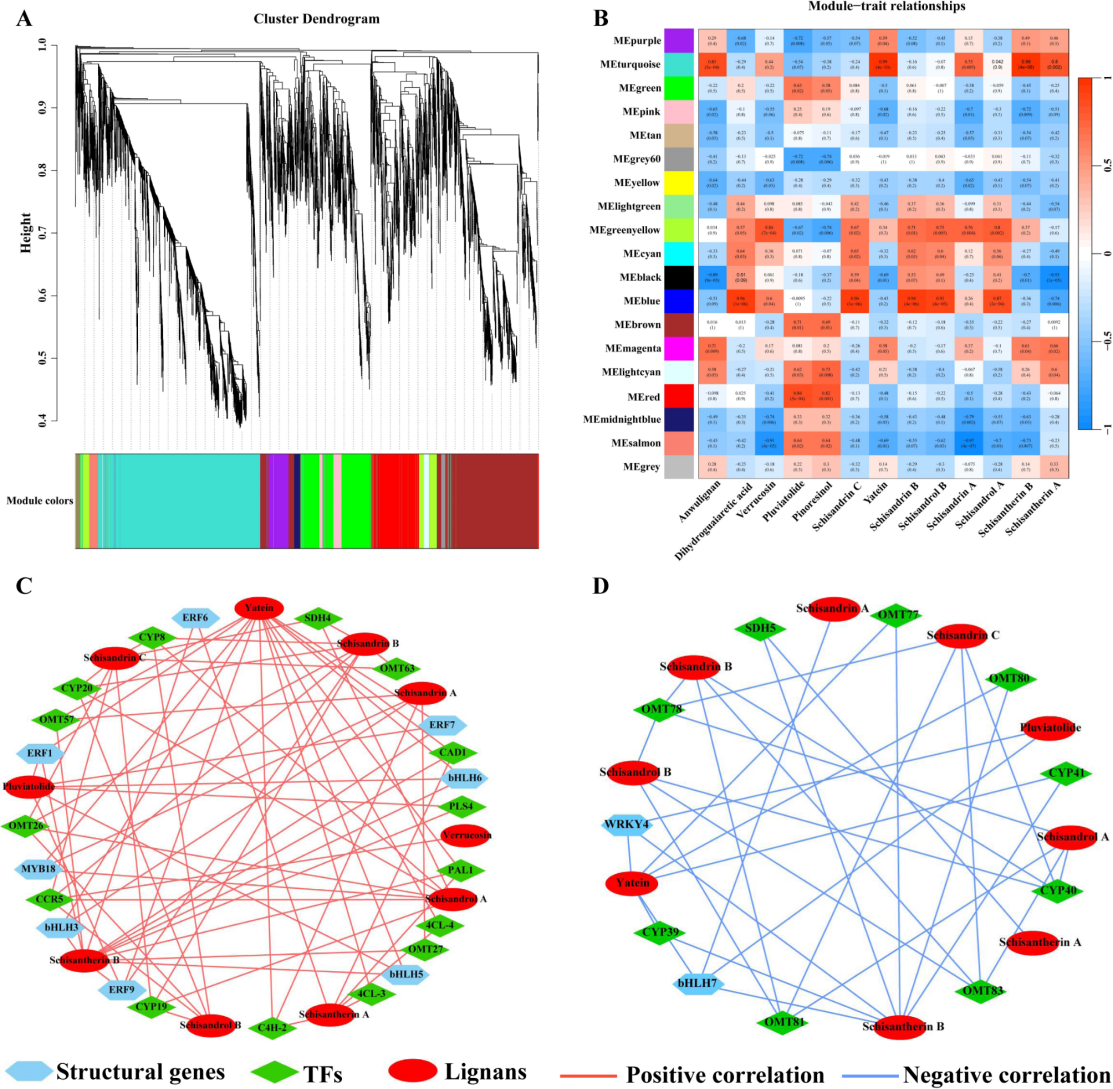
WGCNA co-expression analysis based on differentially expressed genes and lignans in *S. sphenanthera*. (A) The identified gene modules are labeled in different colors. (B) The gene module–trait relationship. (C) The co-expression network of positive correlation. (D) The co-expression network of negative correlation

WGCNA analysis was performed to obtain the genetic regulatory networks of lignan biosynthesis in *S. sphenanthera*. The unigenes were clustered into 19 primary modules, among which turquoise, green-yellow, and brown modules were the most abundant (Fig. 7A). As shown in Fig. 7B and Table. S14, the ME-turquoise (91 genes), ME-green-yellow (11), and ME-blue (113 genes) modules were highly associated with lignans accumulated in *S. sphenanthera*. The content of anwulignan, yatein, schisandrin A, schisantherin A, and schisantherin B exhibited significantly positively correlations with the turquoise module. The content of verrucosin, schisandrin A, schisandrin B, schisandrin C, schisandrol A, and schisandrol B showed significantly positively correlation with the green-yellow module, whereas pluviatolide and pinoresinol exhibited the opposite correlation. The content of dihydroguaiaretic acid, verrucosin, schisandrin B, schisandrin C, schisandrol A, and schisandrol B exhibited significantly positively correlations with the blue module, whereas schisantherin A showed the opposite correlation. The construction of putative genetic and metabolic regulatory networks was based on lignans and candidate hub genes identified within the turquoise, green-yellow, and blue modules (Fig. 7C and D). The accumulation levels of 10 lignans were positively regulated by central genes (PAL1, C4H-2, 2 4CLs, CAD1, PLS4, SDH4, CCR5, 4 OMTs, 3 CYPs, 4 ERFs, 3 bHLHs, and MYB18; Fig. 7C). The accumulating levels of nine lignans were negatively regulated by hub genes (SDH5, 5 OMTs, 3 CYPs, bHLH7, and WRKY4; Fig. 7D).

### Validation in structural genes expression by qRT-PCR

**Fig. 8:**
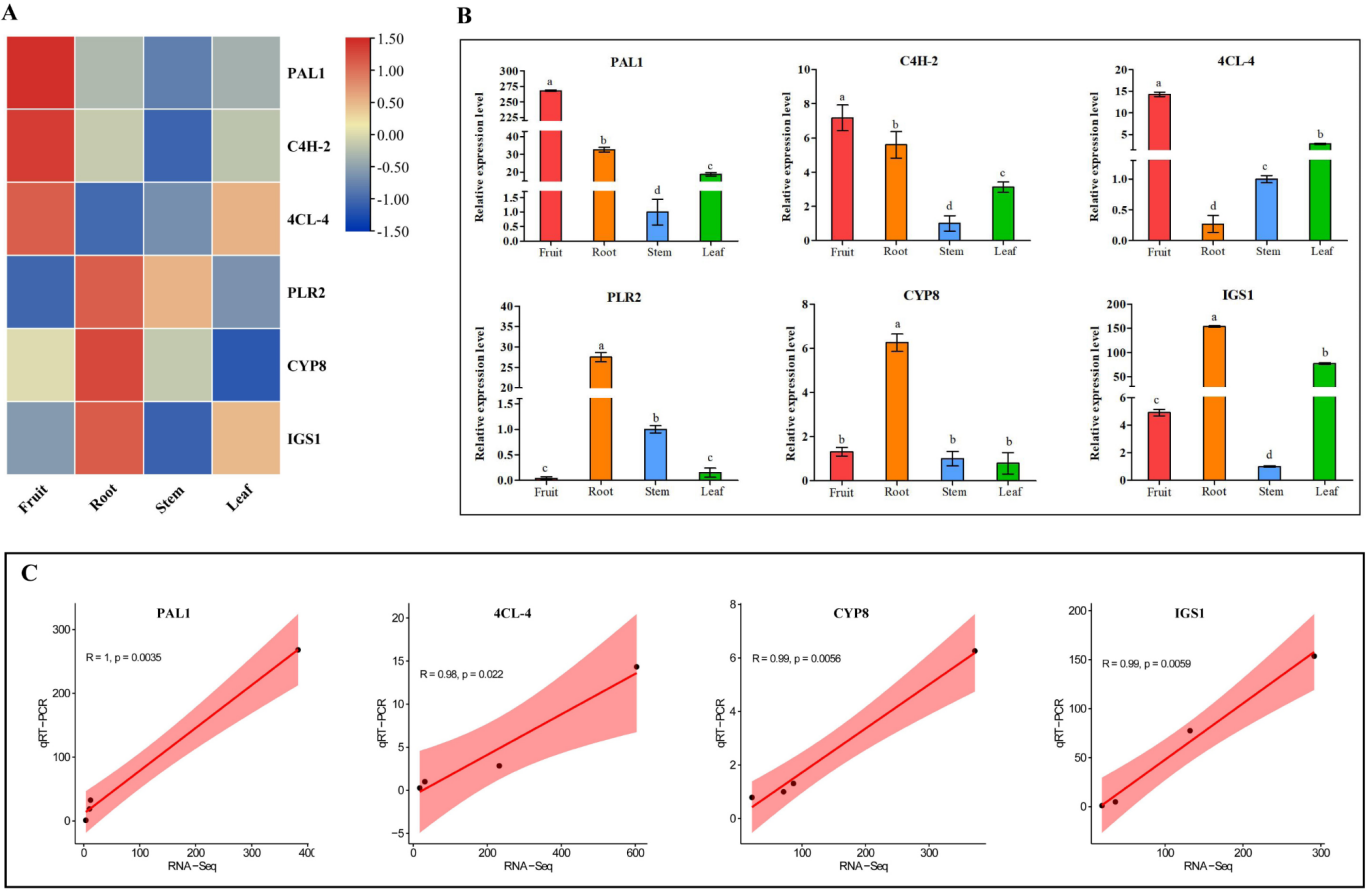
RNA-Seq and qRT-PCR analysis of the structural gene in different tissues including root, stem, leaf and fruit, respectively. (A) Heat map depicting RNA-Seq. (B) qRT-PCR analysis. (C) Scatter plot of Pearson correlation coefficient between RNA-Seq and qRT-PCR.

The structural genes verified by qRT-PCR were obtained by WGCNA analysis and BLAST comparison. Six structural genes related to lignan biosynthesis were selected for qRT-PCR validation to confirm the accuracy and reliability of RNA-seq data. The qRT-PCR results for the selected differentially expressed genes at four tissues showed good correspondence with RPKM values obtained from RNA-Seq, indicating the reliability of RNA-seq data (Fig. 8A and B). Moreover, no significant correlations were found among the relative expression levels of differentially expressed genes (2^−ΔΔCt^ values of qRT-PCR) and RPKM values. The relative expression levels of differentially expressed genes showed significant correlations with RPKM values (RNA-Seq data; p < 0.05; Fig. 8C). The good correspondence between qRT-PCR and RNA-seq data and the significant correlations between their relative expression levels suggested that the RNA-seq data were reliable.

## Discussion

To thoroughly understand lignan biosynthesis in *S. sphenanthera*, we generated data of the transcriptome de novo assembly in the different tissues of *S. sphenanthera* for the first time. In this study, transcriptomes were assembled, annotated, and analyzed in *S. sphenanthera*, resulting in a total of 167,972,229 transcripts and 91,215,760 unigenes (Fig. 1, Tables S1, and Tables S2). To date, only the transcripts of *S. chinensis* fruits have been reported [25, 34, 39, 40], and no study on the draft genome of *Schisandra* plant and transcripts of *S. sphenanthera* has been conducted. In the absence of a reference genome, Trinity, a software tool used for de novo transcriptome assembly, overestimated the number of annotated transcripts and unigenes because it was unable to distinguish between isoforms and alternative splicing events [41]. However, interesting or novel gene transcripts can be identified by this method in the absence of whole genome sequence information [42]. The accuracy of de novo transcriptome assembly is expected to improve with the availability of the well-annotated genomes of *S. sphenanthera*. Therefore, our transcriptome data are valuable references to advance the study of *S. sphenanthera*.

Analysis of gene expression profiling of distinct tissues in *S. sphenanthera* can provide useful information on TSGs, which are genes specifically expressed or highly enriched in a particular tissue. By comparing the gene expression profiles of different tissues, genes that are uniquely expressed in each tissue and those that are commonly expressed across multiple tissues can be identified. This information can be used in exploring the molecular basis of metabolic biosynthesis and tissue development and function and identifying potential targets for improving yield, quality, and adaptation to environmental stress. Therefore, studies that analyze the gene expression profiles of distinct tissues in *S. sphenanthera* can provide valuable insights into the biological process of this species and inform future research efforts.

Fruit and roots have significant specific expression profiles because they are the main sources of bioactive compounds (Fig. 3). To confirm this hypothesis, lignans in different tissues was detected using UPLC–QTOF–MSMS and UPLC–QQQ–MSMS. The data of lignan-targeting metabolism were basically consistent with the gene expression profiles (Figs. 3 and 5). Anwulignan and schisantherin A were used for identifying the *S. sphenanthera* varieties of characteristic substances [5, 6, 43]. This study detected these two substances. Interestingly, high levels of schisandrin B were detected in the roots, stems, leaves, and fruit, which can be used as markers for the quality evaluation of *S. sphenanthera*. In addition, *S. sphenanthera* fruit is commonly used in traditional Chinese medicine and as health food [2], whereas the roots, stems, and leaves are underutilized. In addition to the fruit, the roots were extremely rich in lignans and can be used as the main raw materials for the development of novel drugs, functional food, and other high-value products. The stems and leaves, which can be used to extract specific lignans or processed into feed for full utilization. This study improves people’s understanding of different tissues of *S. sphenanthera* to some extent and can turn waste from the roots, stems, and leaves into valuable products, which have important guiding significance for rational development and application of different tissues of *S. sphenanthera*. Taken together, these results will help to uncover the theoretical basis of different tissues accumulation of lignan biological active products in *S. sphenanthera*.

Understanding the metabolic pathways of natural products can inform efforts to produce these compounds on a larger scale and provide insights for creating novel chemicals using synthetic biology techniques. In this study, the metabolic pathway of lignans, the main bioactive substance in *S. sphenanthera* was deeply analyzed from the molecular point of view, and the phenylpropanoid biosynthetic pathway of lignan biosynthesis was activated in the fruit and roots (Fig. 6). Particularly, the analysis showed that PAL, C4H, 4CL, CCoAOMT, CCR, and CAD genes were up-regulated in the pathway leading to coniferyl alcohol (Fig. 6). In addition, the pathway’ activation was associated with the up-regulation of IGS1 and DIR, which regulate the early steps of lignan biosynthesis (Fig. 6). This result is similar to the study on the lignan biosynthesis of *Isatis indigotica* [44]. The overexpression of PhIGS1 induces isoeugenol accumulation in *Petunia hybrida* [45]. Therefore, the up-regulation of genes related to phenylpropanoid biosynthetic pathway in the fruit and roots can promote lignan accumulation in *S. sphenanthera*. Gene expression can provide important information about the potential production of a metabolite, and it does not always directly correlate with the actual accumulation level of the metabolite. The reason is that post-transcriptional and post-translational regulation mechanisms can affect the stability, activity, and localization of resulting enzymes and ultimately impact the final metabolite levels.

**Fig. 9:**
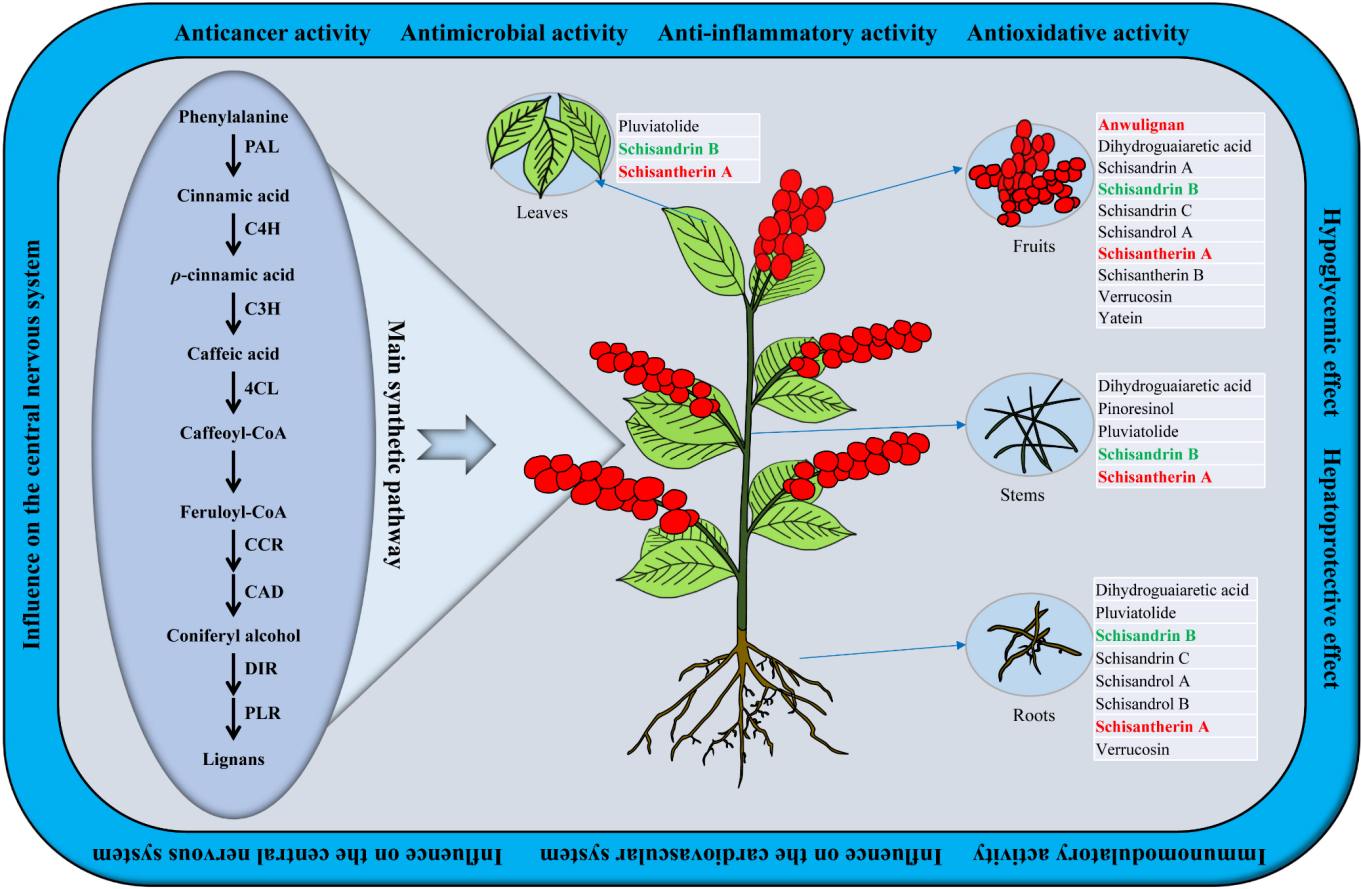
Proposed model of different tissues in lignan accumulation in *S. sphenanthera*

In this study, the transcriptome and metabolome were combined to analyze the gene expression profiles and metabolite accumulation levels in different tissues of *S. sphenanthera*, and the potential application and synthetic models were proposed (Fig. 9). The results showed that the gene expression profiles of different tissues was closely related to the pattern of metabolite accumulation. Through BLAST and WGCNA screening, it was found that the genes related to whole lignan biosynthetic pathway were significantly up-regulated, and this effect was conducive to lignan accumulation. In addition, MYB, bHLH and ERF transcription factors play important regulatory roles in the lignan biosynthetic pathway. For example, TcMYB1, TcMYB4, and TcMYB8 are involved in the positive regulation of lignan biosynthesis in *Taiwania cryptomerioides* Hayata [46]. Among them, TcMYB1, TcMYB4, and TcMYB8 are associated with four MYBs (AtMYB20, AtMYB42, AtMYB43, and AtMYB85), two MYBs (AtMYB46 and AtMYB83), and two MYBs (AtMYB61 and AtMYB50) of *Arabidopsis Thaliana* clustered together in the same branch [46]. As shown in Figure S1, phylogenetic tree analysis revealed 14 MYB transcription factors that may be associated with lignan regulation in *S. sphenanthera*. MYB18 is an important MYB that positively regulates transcription factors obtained by WGCNA screening and is one of the 14 transcription factors analyzed by phylogenetic tree analysis. Therefore, the analysis of structural genes and transcription factors in the biosynthetic pathway has a positive effect that promotes the accumulation of bioactive substances.

## Conclusion

Obtaining the expression patterns of TSGs and metabolite biosynthesis–related genes is a foundation for future studies in metabolomics and functional genomics. The transcriptome and metabolome data provide useful resources for the study of other genes involved in lignan biosynthesis in *S. sphenanthera*. This dataset can provide a reference for the follow-up studies on lignan metabolism, molecular identification, and molecular breeding.

### Electronic supplementary material

Below is the link to the electronic supplementary material.

Supplementary Material 1

Supplementary Material 2

## Data Availability

The datasets generated and/or analysed during the current study are available in the NCBI repository, number: PRJNA971086 (https://dataview.ncbi.nlm.nih.gov/object/PRJNA971086?reviewer=q407aplnpastl84f573t73rnmo).
