# Supplementary figures and images for: Tissue-specific transcriptome and metabolome analyses reveal candidate genes for lignan biosynthesis in the medicinal plant Schisandra sphenanthera
Source: BMC Genomics. 2023 Oct 11;24:607. doi: 10.1186/s12864-023-09628-3 (PMC10568845; doi:10.1186/s12864-023-09628-3)

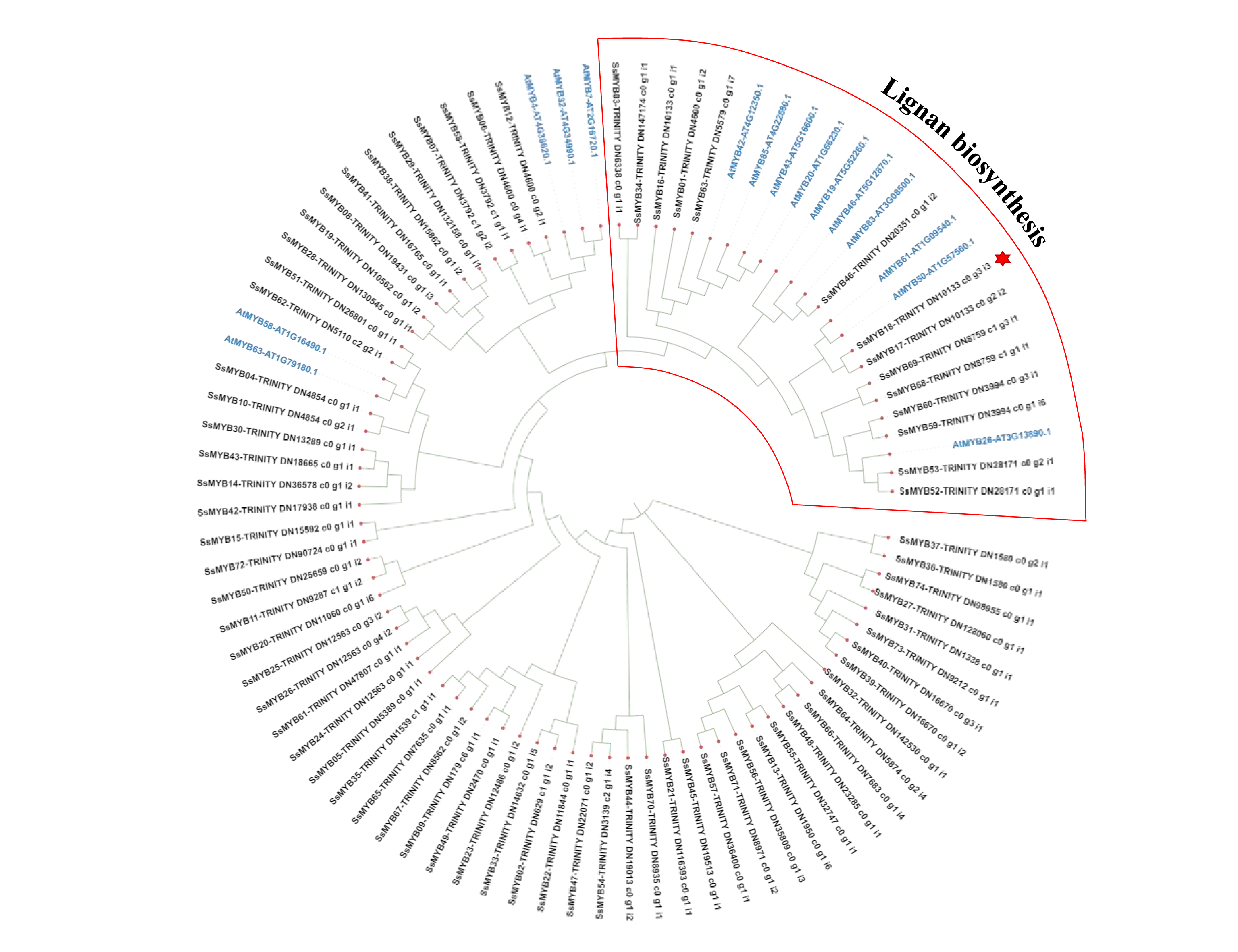

Supplement: Supplementary file 1 — Supplementary Material 1 [file 12864_2023_9628_MOESM1_ESM.tif]
